# Pilot study of docetaxel combined with lobaplatin or gemcitabine for recurrent and metastatic breast cancer

**DOI:** 10.1097/MD.0000000000018513

**Published:** 2019-12-27

**Authors:** Fenghu Li, Bi Wang, Mingyuan He, Jianying Chang, Jiehui Li, Lang Shan, Heran Wang, Wei Hong, Daiqin Luo, Yang Song, Liyang Liu, Huiqin Li, Li Ran, Tengxiang Chen

**Affiliations:** a Department of Breast and Gynecologic Oncology, Affiliated Hospital of Guizhou Medical University; b Department of Breast and Gynecologic Oncology, Guizhou Cancer Hospital; c Department of Obstetrics and Gynecology, Guiyang Maternal and Child Health-Care Hospital; d Guizhou Provincial Key Laboratory of Pathogenesis and Drug Research on Common Chronic Diseases; Guizhou Province Key Laboratory for Regenerative Medicine; Department of Physiology, School of Basic Medicine, Guizhou Medical University, Guiyang, Guizhou, China.

**Keywords:** combined chemotherapy, curative effect, docetaxel, gemcitabine, lobaplatin, recurrent metastatic breast cancer

## Abstract

**Background:**

This study evaluated the efficacy and safety of docetaxel combined with lobaplatin, relative to docetaxel combined with gemcitabine, for treating patients with recurrent metastatic breast cancer (rMBC).

**Methods:**

Patients with rMBC received ≥2 cycles (21 days each) of either docetaxel and lobaplatin (DL; *n* = 21), or docetaxel and gemcitabine (DG; *n* = 22). On day 1 of each cycle, all patients were given 75 mg/m^2^ intravenous docetaxel. Patients in DL and DG were also given, respectively, 35 mg/m^2^ intravenous lobaplatin (day 2) or 1000 mg/m^2^ intravenous gemcitabine (days 1, 8).

**Results:**

Five (11.6%) and 16 (37.2%) patients achieved complete remission and partial response, respectively; rates of response and disease control were 48.8%. The response rates of the groups were comparable (47.6%, 50.0%). The median survival times after relapse and metastasis of the DL group (18 months) were significantly less than that of the DG group (25 months). Median progression-free survivals after relapse and metastasis were similar (12 cf. 14 months). The main toxic side reaction was grade 2, with no treatment-related deaths. Rates of the following were comparable between DG and DL: grade 3 or 4 white blood cells (23.8%, 31.8%) and digestive tract toxicity (4.8%, 4.5%); neutropenia (28.6%, 22.7%); anemia (4.8%, nil); and thrombocytopenia (19.0%, 13.6%). Other toxicities included hepatic toxicity, myalgia, infection, and fatigue.

**Conclusions:**

Both the DL and DG regimens were associated with encouraging benefits, while treatment-related toxicity was manageable. Therefore, these regimens are effective options for treatment of rMBC.

**Trial registration:**

This clinical trial study was approved by the Ethics Committee of Guizhou Cancer Hospital, and has been registered in the China Clinical Trial Center (December 8, 2014, No. ChiCTR-IPR-14005633).

## 1 Introduction

Breast cancer remains the most common malignant tumor in women, despite improvements in diagnosis and treatment. Even when treated in the early stage, nearly 50% of patients with breast cancer still experience relapse and metastasis,^[1]^ with significantly worse prognosis. For these patients, systematic and comprehensive treatment is required, including systemic chemotherapy, endocrine therapy, targeted molecular therapy, and local palliative treatment.^[2]^

Systemic chemotherapy is one of the most effective treatment options for recurrent metastatic breast cancer (rMBC), although there is no gold standard regimen. Many chemotherapy regimens include anthracycline combined with Taxus drugs. However, the clinical application of anthracycline is restricted by cumulative cardiac toxicity and drug resistance.^[3]^

Docetaxel has been recommended as a first-line drug for rMBC.^[4]^ Some pre-clinical trials have found that platinum has good anti-tumor activity in breast cancer, especially in triple-negative breast cancer.[^5^,^6^] However, the effective rate of platinum-based cisplatin in recurrent and metastatic breast cancer is only 6% to 20%, either alone or in combination.[^7^,^8^] Lobaplatin ([1,2-diamino-methylcyclobutane] platinum [II]-lactate) is a dual small-molecule inhibitor of EGFR (epidermal growth factor receptor and HER2 (human epidermal growth factor receptor 2) without cross resistance with cisplatin, and the combined regimen is effective in breast and lung cancer.^[9]^ In addition, the pyrimidine nucleoside antimetabolic drug gemcitabine has been shown to have a certain effect in rMBC.^[10]^

There is a lack of clinical study of docetaxel combined with lobaplatin, or of docetaxel combined with gemcitabine. To this end, the present pilot clinical study was conducted to compare the efficacy and side effects of the 2 schemes in rMBC.

## 2 Methods

This clinical trial study was approved by the Ethics Committee of Guizhou Cancer Hospital, and has been registered in the China Clinical Trial Center (ChiCTR-IPR-14005633). All the patients provided signed informed consent before treatment.

### 2.1 Patients

The patients conformed to the following eligibility criteria: aged ≥18 years; with histologically confirmed rMBC; 6 months since last chemotherapy; 2 weeks since endocrine therapy; expected survival time ≥3 months; Eastern Cooperative Oncology Group (ECOG) performance status score 0-1; and no apparent abnormalities in routine blood test, liver, or kidney function. The latter were defined as follows: hemoglobin ≥9 g/dL; absolute neutrophil count ≥1500/μL; platelets ≥100,000/μL; total bilirubin <1.5-fold the institutional upper limit of normal (ULN); aspartate aminotransferase and alanine aminotransferase ≤2.5 ULN, or <5 ULN if hepatic metastases are present; and creatinine ≤1.5 ULN.

Patients with any of the following were excluded from this study: history of allergies to chemotherapeutic drugs or drug additives; or severe complications such as renal insufficiency, severe infection, or mental illness caused by diabetes. Other grounds for exclusion were: pregnant or breast-feeding; extensive liver metastasis or pulmonary metastasis with dyspnea; brain metastasis with symptoms; uncontrolled hypertension, angina, or congestive heart failure; pulmonary fibrosis; or interstitial pneumonia.

### 2.2 Trial design

Patients were assigned the specific chemotherapy regimen by self-selection of sealed, opaque envelopes containing the regimen generated by computerized random distribution sequence. Eligible subjects opened the envelopes only after agreeing to enter the test and accept the corresponding treatment scheme. The patients were thus assigned to receive either docetaxel and lobaplatin (DL) or docetaxel and gemcitabine (DG).

The primary efficacy parameter was the best overall response by investigator's assessment, in accordance with the Response Evaluation Criteria in Solid Tumors (RECIST, version 1.1).^[11]^ Complete response (CR) was defined as disappearance of all target lesions. Partial response (PR) was a ≥30% decrease in the sum of the longest diameter of target lesions, from the baseline (at least one measurable lesion). Progressive disease was considered a ≥20% increase in the sum of the longest diameter of target lesions or the appearance of ≥1 new lesion. Stable disease was considered as neither a sufficient reduction to qualify for PR, nor a sufficient increase to qualify for progressive disease. Toxicity was assessed using the Common Terminology Criteria for Adverse Events (version 3.0).

The primary objective of the study was to determine the effectiveness of the DL and DG schemes (effectiveness defined as CR + PR) and toxicity. The secondary endpoint was the survival time (defined as the time from the beginning of group assignment to death due to any cause or the last follow-up). Progression-free survival was the time from the beginning of group assignment to tumor progression or death or last follow-up.

### 2.3 Treatment and dosage regimens

**Figure 1:**
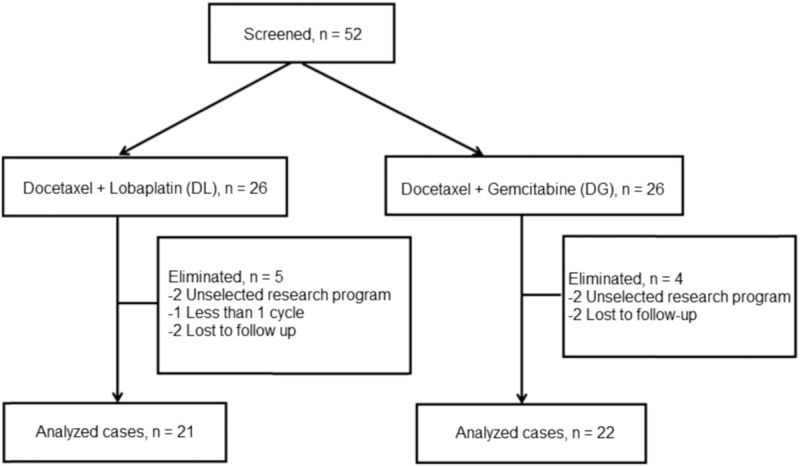
Flow diagram of project study.

On day 1 of each cycle, all patients were given 75 mg/m^2^ intravenous docetaxel (Fig. 1). Specifically, oral dexamethasone (16 mg) was given at 12, 8, and 1 hour before docetaxel was infused. Docetaxel (20 mg/ampoule; Jiangsu Hengrui Pharmaceutical, China; 75 mg/m^2^, dissolved in 250 mL sodium chloride injection) was administered by venoclysis >1 hour on the first day of each 21-day cycle. Two and three days after docetaxel infusion, dexamethasone was given (8 mg/time), twice per day.

#### 2.3.1 DL group

In addition to docetaxel (described above), patients in the DL group received lobaplatin (10 mg/ampoule; Hainan Changan International Pharmaceutical, China; 35 mg/m^2^, dissolved in 250 mL sodium chloride injection) by venoclysis on the second day of each 21-day cycle (Fig. 1). Treatment was continued to progression or intolerable toxicity, or refusal to continue treatment.

#### 2.3.2 DG group

In addition to docetaxel (described above), patients in the DG group received gemcitabine (Jiangsu Hengrui Pharmaceutical, China; 1000 mg/m^2^) by intravenous drip >30 minutes, on days 1 and 8 of each 21-day cycle (Fig. 1). Treatment was continued to progression or intolerable toxicity, or refusal to continue treatment.

### 2.4 Statistical methods

All data were analyzed using SPSS (Version 19.0) software. Categorical variables were analyzed with the chi-squared (*χ* ^2^) test. Patient survival curves were estimated by the Kaplan–Meier method. A *P* value <.05 was considered statistically significant.

## 3 Results

### 3.1 Patients’ general data

From 1 January 2014 to 6 March 2016, 52 patients with rMBC, and who conformed to the inclusion criteria for this study, were admitted to the Department of Breast and Gynecologic Oncology of Guizhou Provincial Cancer Hospital. Of these, 4, 4, and 1 were excluded, respectively due to missed visit, without study regimen, and only 1 cycle of chemotherapy.

Therefore, the present study comprised 43 women, aged 27 to 70 years (median, 47 years; Table 1). More than 50% of the patients were found to have visceral metastasis. The DL and DG groups consisted of 21 and 22 patients, respectively. The general data of the 2 groups were statistically similar. The median number of chemotherapy cycles in both the groups was 4 (2–6 cycles; Table 2).

**Table 1:**
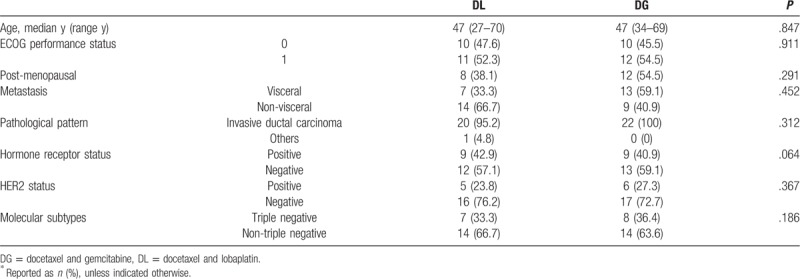
Baseline characteristics of patients^∗^.

**Table 2:**
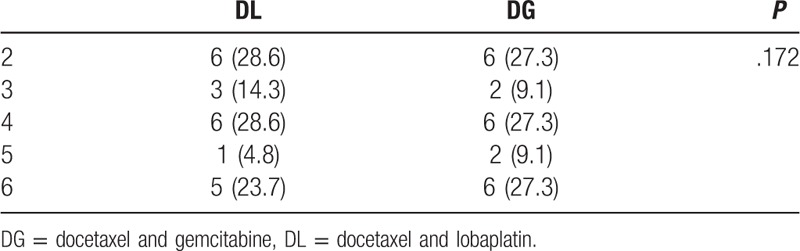
Completion of treatments by cycles of chemotherapy, *n* (%).

### 3.2 Immediate curative effect of the 2 groups

The rates of CR, PR, and treatment effectiveness (CR + PR) of the 2 groups were statistically comparable (Table 3). Specifically, in the DL (DG) groups there were 3 (2) cases of CR, and 7 (9) cases of PR. The rates of CR + PR of the DL and DG groups were 47.6% and 50.0%.

**Table 3:**
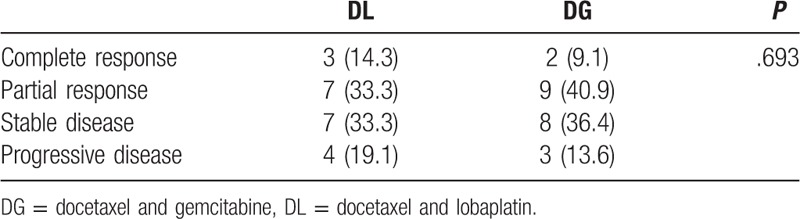
Tumor treatment response rates, *n* (%).

### 3.3 Patient's survival time

**Figure 2:**
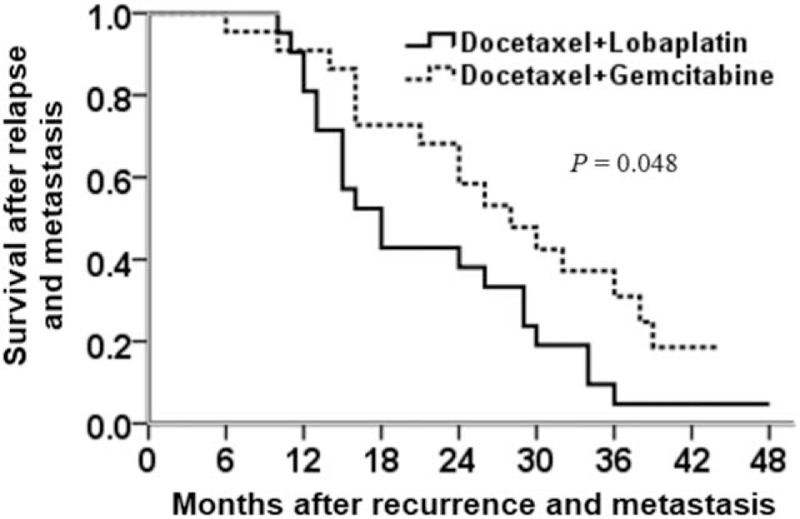
Comparison of survival between the docetaxel and lobaplatin and docetaxel and gemcitabine groups.

All patients were followed until 31 October 2018 (Fig. 2). Overall, there were 36 deaths, with 20 and 16 deaths in the DL and DG groups, respectively. The median survival time from baseline (defined as the time from the beginning of group assignment to the last follow-up) was 24 months (6–48 months). The 18-month (10–48 months) survival of the DG group was slightly worse than that of the 25-month (6–44 months) survival of the DL group (*P* = .048).

**Figure 3:**
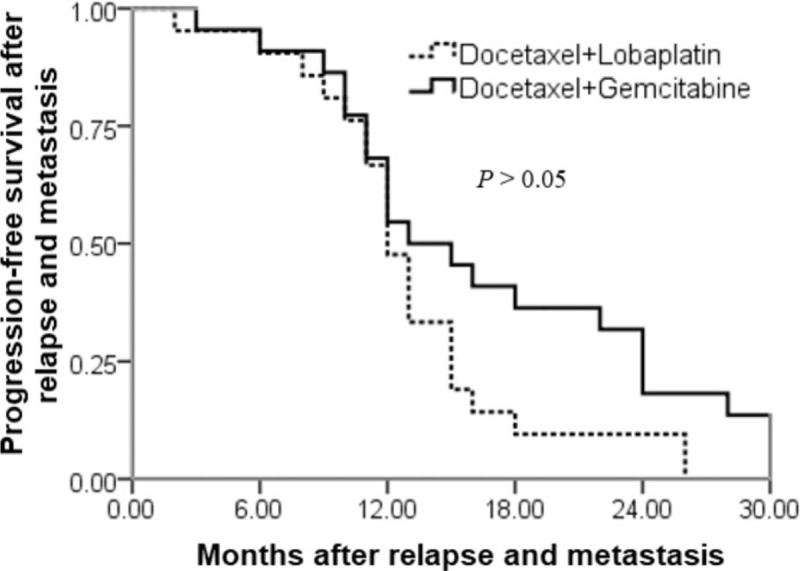
Comparison of progression-free survival between the docetaxel and lobaplatin and docetaxel and gemcitabine groups.

The median progression-free survival time from progression, recurrence, and metastasis to progression of the DL and DG groups were statistically similar (Fig. 3). Specifically, in the DL group the median survival time from progression, recurrence, and metastasis to progression in 21 cases was 12 months (2–26 months). In the DG group the median survival time from progression, recurrence, and metastasis to progression in 22 cases was 14 months (3–30 months).

### 3.4 Treatment related toxic and side effects

There were no deaths related to treatment in either of the groups (Table 4). The major side effects associated with treatment were grade 2 toxic side reaction. The 2 groups were statistically comparable in rates of toxicity and side effects. Regarding bone marrow suppression, the grade 3 or 4 reactions of white blood cells, neutrophil granulocytes, hemoglobin, platelets, and digestive tract in the DL (DG) groups were, respectively, 23.8% (31.8%), 28.6% (22.7%), 4.8% (nil), 19.0% (13.6%), and 8% (4.5%). The rates of hepatic toxicity, pain, infection, and fatigue in the DL (DG) groups were 0% (4.5%), 4.8% (4.5%), 4.8% (nil), and 9.5% (13.6%).

**Table 4:**
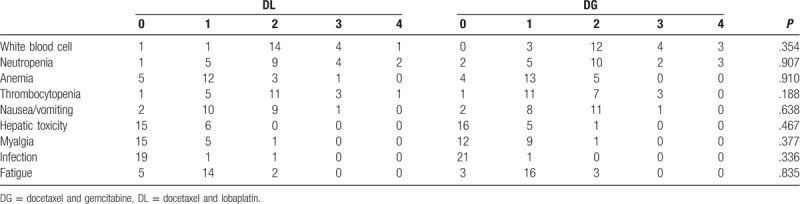
Treatment-related clinical adverse events according to cycle of chemotherapy.

## 4 Discussion

With advances in surgery, radiotherapy, chemotherapy, endocrinology, and targeted therapy, the survival rate of breast cancer patients has improved significantly. However, ∼30% of patients with early breast cancer develop recurrence and metastasis within 5 years after surgery.^[12]^ Almost 90% of deaths due to breast cancer are caused by tumor metastasis, and nearly 80% of patients died within 1 year after receiving a diagnosis of recurrent and metastatic breast cancer.^[13]^ Therefore, recurrent and metastatic breast cancer is the leading cause of death in women.^[14]^ The purpose of treatment of recurrent and metastatic breast cancer is palliative care to improve quality of life, and reduce tumor-related complications.[^15^,^16^] Treatment should be both safe and effective. There remain challenges to the treatment strategy for advanced breast cancer, and there is a lack of expert consensus^[17]^ on management strategies.[^18^,^19^] Systemic chemotherapy is relatively effective to relieve the disease, with rates of therapeutic effectiveness of 11.1% and 51.9% for single-drug and combined regimens, respectively. Yet, for recurrent and metastatic breast cancer chemotherapy, there is no standard protocol. New drugs and chemotherapies require testing in clinical trials for application in recurrent and metastatic breast cancer.

As a cell-cycle specific drug, docetaxel stabilizes intracellular microtubules, induces the assembly of microtube bundles, and inhibits cell proliferation and division, blocking cells in M phase.^[20]^ A meta-analysis^[21]^ showed that a 3-week treatment regimen of docetaxel improved overall survival, disease progression time, and the clinical prognostic factors of advanced breast cancer.^[4]^ Thus, docetaxel was recommended for the treatment of advanced breast cancer, and as a first-line drug for rMBC.

Lobaplatin, a cell-cycle nonspecific drug, is a third-generation platinum antitumor compound developed by ASTA Medica. The mechanism involves the formation of bonds between platinum and nitrogen atoms in the DNA base, leading to crosslinking and torsion, which inhibits the function of the tumor DNA.^[9]^ Clinical studies have shown that the anticancer treatment index of lobaplatin is equal to or higher than that of cisplatin and carboplatin. It is effective for partial cisplatin-resistant and carboplatin-resistant tumors, with no obvious renal toxicity, neurotoxicity, or ototoxicity, and its digestive tract reaction is less than that of cisplatin. Thrombocytopenia limits the dose, similar to carboplatin,^[22]^ and thus it is used for the treatment of cancers of the lung, breast, cervix, and others.[^23^,^24^,^25^,^26^,^27^]

Gemcitabine is a cell-cycle specific pyrimidine antimetabolic drug. The major role of gemcitabine is in tumor cell DNA synthesis, that is, in S phase.^[28]^ The effective rate regarding recurrence and metastasis of breast cancer is ∼20%, with good tolerance. Gemcitabine is the first-line chemotherapy drug for advanced breast cancer recommended by the China Anticancer Association.^[29]^

Although there are many chemotherapeutic regimens for recurrent and metastatic breast cancer, the therapeutic effects of only a few have been reported. Yang et al^[30]^ investigated lobaplatin combined with vinorelbine for treatment of advanced breast cancer, and reported rates of effectiveness, disease control, grade 3 or 4 white blood cells, and platelet decline of 39.1%, 76.1%, 45.7%, and 8.7%, respectively, and mild non-hematological toxicity. He et al^[31]^ observed docetaxel combined with gemcitabine in the treatment of advanced breast cancer, and found the scheme to be effective, with mild adverse effects. However, to our best knowledge reports of comparisons of the 2 schemes are few.

The present study is the first comparison of the DL and DG schemes in recurrent and metastatic breast cancer in which the efficacy and safety of these regimens were observed. The general clinical data of the patients in the 2 groups were comparable. Overall, 179 cycles of systemic chemotherapy were administered to the patients, with a median of 4 cycles (2–6) per patient and 4 cycles (2–6) in each of the 2 groups. The immediate curative effect reported in this study is consistent with the results reported in the literature,[^28^,^29^,^30^,^31^,^32^] in which the overall efficiency of treatment was 48.8% (5 and 16 cases of CR and PR, respectively, among 43 patients). Furthermore, the CR + PR rates of effectiveness of the DL (47.6%; 3 and 7 cases of CR and PR) and DG (50.0%; 2 and 9 cases of CR and PR) groups were similar.

At the last follow-up, overall 36 (83.7%) patients had died, and median survival time from recurrence and metastasis to the last follow-up was 24 months. The median survival time of the DG group (25 months; with 72.7% or 16 deaths) was marginally but significantly better than that of the DL group (18 months; with 95.2% or 20 deaths).

The median progression-free survival time after recurrence and metastasis in the entire group was 13 months, which is better than the results reported in the literature (8–12 months).[^31^,^32^] The DL and DG groups of the present study were comparable regarding rates of progression (95.2% and 95.5%, respectively) and median time to progression (12 and 14 months). These results suggest that the DL and DG regimens are effective in recurrent and metastatic breast cancer, especially with regard to disease-free survival time.

When patients with recurrent and metastatic breast cancer have been treated with multiple chemotherapy cycles and regimens, their general condition is poor, especially with regard to bone marrow hematopoiesis and liver and kidney function. It is urgent to find effective chemotherapy protocols with low toxicity. The DL and DG regimens evaluated in the present study were effective, with treatment-related toxicity of mainly grade 2, and no treatment-related death occurred.

The 2 groups were similar in bone marrow suppression, with rates of grade 3 or 4 white blood cells, neutrophil granulocytes, hemoglobin, and platelets in the DL (DG) groups of 23.8% (31.8%), 28.6% (22.7%), 4.8% (nil), and 19.0% (13.6%), respectively. There were also no differences in the rates of digestive tract grade 3 or 4 reactions in the DL (4.8%) and DG (4.5%) groups, or in the rates of grade 2 liver toxicity, pain, infection, and fatigue, which were nil (4.5%), 4.8% (4.5%), 4.8% (nil), and 9.5% (13.6%). Toxic side effects were mainly leukopenia, granulocyte, thrombocytopenia, and fatigue. In the main, patients tolerated and successfully completed chemotherapy. During the treatment, no patient terminated treatment due to toxic side effects.

In summary, in this study the efficacy and safety of the DL and DG regimens for treatment of recurrent and metastatic breast cancer were observed for the first time. Based on the results, both of these combined chemotherapy regimens are effective, treatment-related side effects are tolerable, and either can be used for effective treatment for advanced breast cancer. The results of the study may be affected by the small sample size and short follow-up time. The results of this pilot study warrant further verification in clinical trials with larger sample sizes and longer follow-up times.

## Author contributions

**Project administration:** Bi Wang, Jianying Chang, Jiehui Li, Lang Shan, Wei Hong, Huiqin Li.

**Resources:** Mingyuan He.

**Writing – original draft:** Fenghu Li.

**Writing – review & editing:** Li Ran, Tengxiang Chen.
